# Oncogenic PI3K/AKT promotes the step-wise evolution of combination BRAF/MEK inhibitor resistance in melanoma

**DOI:** 10.1038/s41389-018-0081-3

**Published:** 2018-09-20

**Authors:** Mal Irvine, Ashleigh Stewart, Bernadette Pedersen, Suzanah Boyd, Richard Kefford, Helen Rizos

**Affiliations:** 1 Faculty of Medicine and Health Sciences, Macquarie University, Sydney, NSW Australia; 2 Melanoma Institute Australia, Sydney, NSW Australia

## Abstract

Nearly all patients with BRAF-mutant melanoma will progress on BRAF inhibitor monotherapy and combination BRAF/MEK inhibitor therapy within the first year of therapy. In the vast majority of progressing melanomas, resistance occurs via the re-activation of MAPK signalling, commonly via alterations in BRAF, NRAS and MEK1/2. A small proportion of resistant melanomas rely on the activation of the compensatory PI3K/AKT signalling cascade, although activation of this pathway does not preclude patient responses to BRAF/MEK inhibition. We now show, that PI3K/AKT signalling via potent oncogenic PIK3CA and AKT3 mutants, is not sufficient to overcome proliferative arrest induced by BRAF/MEK inhibition, but rather enables the survival of a dormant population of MAPK-inhibited melanoma cells. The evolution of resistance in these surviving tumour cells was associated with MAPK re-activation and no longer depended on the initial PI3K/AKT-activating oncogene. This dynamic form of resistance alters signalling dependence and may lead to the evolution of tumour subclones highly resistant to multiple targeted therapies.

## Introduction

Acquired resistance to BRAF inhibitor monotherapy and combination BRAF/MEK inhibition occurs in most patients with metastatic BRAF^V600E/K^-mutant melanoma, and only 20% of patients on combination treatment remain progression free at 3 years^1^. In 50–75% of melanomas progressing on BRAF inhibitor or combination BRAF/MEK inhibition, common resistance effectors, including *BRAF* copy number gains, *MEK1/2* and *NRAS* mutations promote the reactivation of MAPK signalling^2–6^. A further 20% of BRAF inhibitor resistant melanomas acquire alterations that stimulate both the MAPK cascade and the compensatory PI3K/AKT survival network. These alterations include gain of function NRAS and KRAS mutations and overexpression of receptor tyrosine kinases, including EGFR, PDGFRß, and MET^2,5^. An additional 4% of BRAF inhibitor resistant melanomas display genetic alterations, including oncogenic AKT1/3 mutations, loss of function PTEN mutations and putative functional mutations in other PI3K regulatory genes (i.e. *PIK3CA*, *PIK3CG*, *PHLPP1* and *PIK3R2*) that activate only the PI3K/AKT network^5^.

Although acquired activation of PI3K signalling occurs in a subset of melanomas it remains unclear whether PI3K activation alone is sufficient for the development of BRAF inhibitor resistance. For instance, baseline expression of phosphorylated AKT, a downstream marker of PI3K signalling, is not associated with response to BRAF inhibition^7^. Similarly, loss of PTEN, a negative PI3K/AKT regulator and negative prognostic factor for melanoma, is associated with shorter progression free survival in BRAF inhibitor-treated patients but is not predictive of best overall response^8,9^. Complete and partial responses to BRAF inhibition have been observed in patients with complete loss of PTEN tumour expression and with PIK3CA tumour-associated activating mutations^7,9,10^. Further, although loss of NF1 and PTEN in melanoma cells is associated with PI3K/AKT activation, these cells remain dependent on MAPK signalling, rather than PI3K/AKT activity for proliferation^11,12^ and this coincides with equivalent response rates in NRAS-mutant and BRAF-mutant melanoma patients treated with the MEK inhibitor binimetinib^13^. Finally, although BRAF^V600E^-mutant melanomas can display an early adaptive response to BRAF inhibition that results in increased PI3K/AKT activity^5^, the majority of resistant tumours display MAPK signalling reactivation^4^.

In this report, we explored the precise contribution of PI3K/AKT activity in melanoma responses to combination BRAF and MEK inhibitor treatment. We reviewed cell line response data and confirmed that both oncogenic BRAF and NRAS predict sensitivity to MEK inhibition in melanoma. We show that PI3K/AKT activating mutations in melanoma do not enable proliferative escape in response to combination BRAF and MEK inhibition, but rather allow the survival of a dormant sub-population of MAPK-inhibited tumour cells, that remain under selective pressure to reactivate MAPK signalling. This is supported by in vivo human data showing that BRAF-mutant melanoma tumours with AKT signalling pre-treatment can undergo substantial tumour shrinkage in response to combination BRAF/MEK inhibition^3^. The persistence of dormant tumour cells under selective pressure will drive the reorganisation of signalling circuits^14^, and this may have critical implications for the evolution of resistance, which in our PI3K/AKT-activated melanoma cell models, involved the complex de-differentiation pathway. This model of acquired resistance is highly resistant to multiple targeted therapies and responds only partially to combination PI3K/MAPK inhibition. This is significant for first-line therapy selection, as the survival of dormant MAPK-inhibited tumour cells may accelerate the evolution of highly resistant tumour subclones with altered signalling dependence.

## Results

### PI3K/AKT activation does not preclude response to BRAF and MEK inhibition

**Fig. 1:**
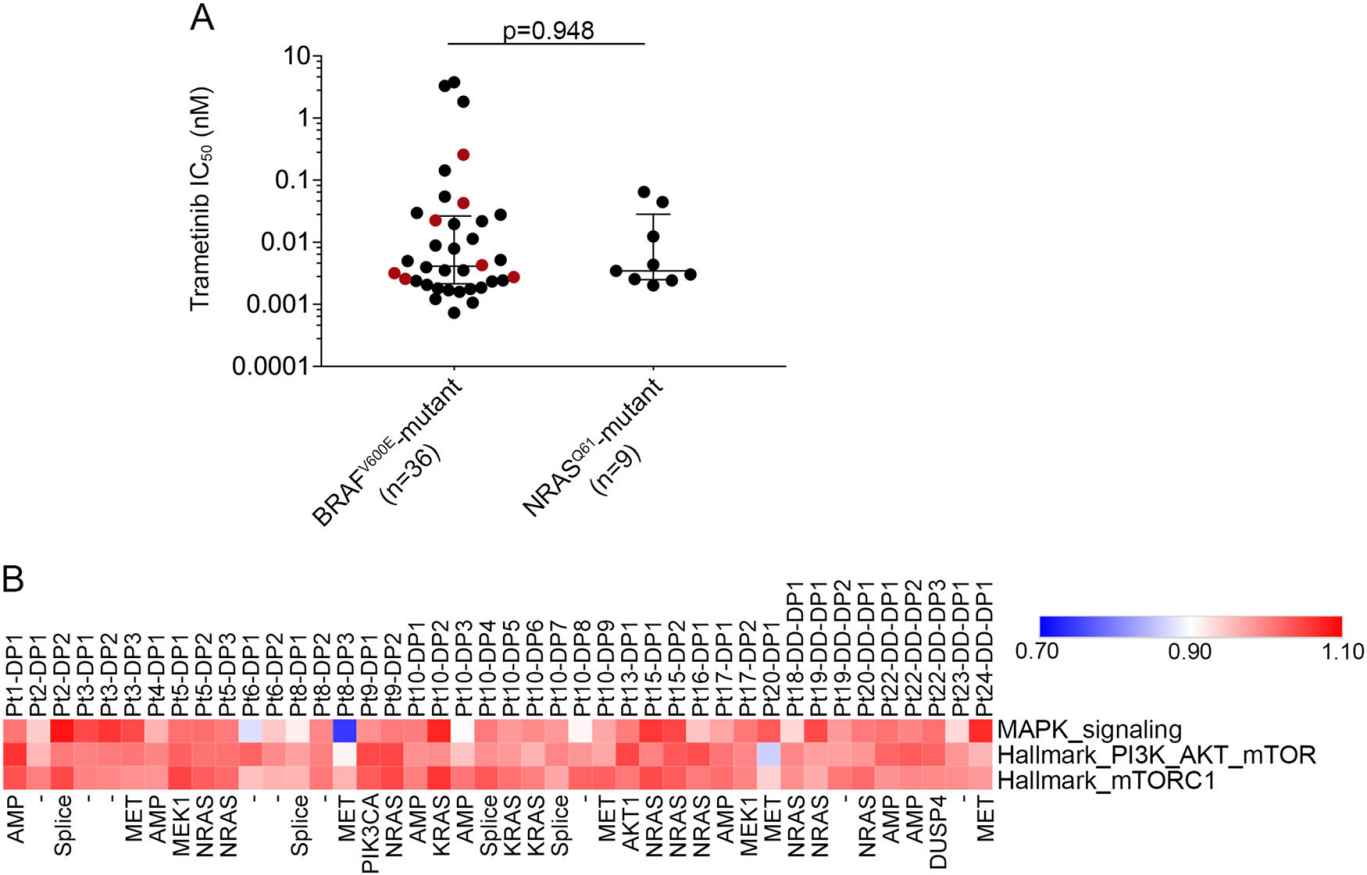
Activation of PI3K by RAS mutations does not diminish sensitivity of melanoma cells to MEK inhibition. **a** Trametinib sensitivity data (IC_50_ values) derived from the Genomics of Drug Sensitivity in Cancer database comparing BRAF^V600E^-mutant and NRAS^Q61^-mutant melanoma cell lines. Data highlighted in red indicate BRAF^V600E^-mutant cells with concurrent loss of function NF1, PTEN mutations or the activating NRAS^Q61K^ mutation. The sensitivity of these cells (red points) were not significantly different from the other BRAF^V600E^ mutant melanoma cells (data highlighted in black, Mann–Whitney *p* = 0.410). Two-tailed Mann–Whitney test was used to determine differences between BRAF and NRAS mutant melanoma populations (*p* = 0.948). Median and interquartile ranges are shown on the scatter plot. **b** Heat map showing ssGSEA ratio scores of MAPK^32^, PI3K and mTORC signalling activity in 42 PROG melanomas relative to their matched PRE tumours. The main resistance mechanism is shown below each PROG tumour. Data are derived from^4,5,16,33^ and tumour samples labelled according to^16^. DP single-drug (BRAF inhibitor) PROG tumours, DD-DP double-drug (combination BRAF and MEK inhibitor) PROG tumours. AMP *BRAF* amplification, splice BRAF-splice variant, - unknown mechanism of resistance

To explore the role of PI3K/AKT activation on cancer cell sensitivity to BRAF and MEK inhibition, we initially reviewed the association of mutations known to activate PI3K/AKT signalling (i.e. oncogenic NRAS and KRAS mutations^15^), with drug sensitivity data of more than 900 cancer cell lines in the Genomics of Drug Sensitivity in Cancer (GDSC) database. We compared cancer cell responses to the selective BRAF^V600E/K^-inhibitor dabrafenib and two allosteric MEK inhibitors, trametinib and CI-1040. As expected, mutant BRAF was a strong predictor of BRAF inhibitor sensitivity whereas, mutant BRAF, NRAS and KRAS were all strong predictors of sensitivity to MEK inhibition (Table S1). We also examined trametinib sensitivity of NRAS^Q61^-mutant versus BRAF^V600E^-mutant melanoma cell lines included in the GDSC database and found that both melanoma genotypes were equally responsive to trametinib (Fig. 1a). Similarly, loss of function mutations in the negative PI3K regulators, NF1 and PTEN or activating NRAS mutations, did not significantly reduce the sensitivity of BRAF^V600E^-mutant melanoma cells to trametinib (Fig. 1a). Although, we did not identify any melanoma cell lines with *PIK3CA* activating mutations in the GDSC database, these data indicate that activating RAS mutations and loss-of-function NF1 or PTEN mutations do not diminish the sensitivity of melanoma cell lines to MEK inhibition in vitro.

Next, we explored the timing and frequency of mutations known to activate PI3K/AKT signalling in the development of BRAF/MEK inhibitor resistance in vivo. We identified five melanoma patients with pre-existing tumour mutations known to activate PI3K/AKT signalling. These PI3K/AKT activating mutations included hot spot RAC1 and AKT3 mutations and loss of function PTEN alterations (Table S2). In all five patients, additional MAPK-activating alterations, including NRAS and MEK2 mutations, were acquired in the matched tumours progressing on BRAF inhibitor monotherapy or BRAF/MEK inhibitor combination treatment (Table S2). Importantly, the pre-treatment PI3K/AKT-activating mutations were retained in the progressing tumours. These data suggest that pre-existing PI3K/AKT activating alterations were not sufficient to confer BRAF and MEK inhibitor resistance in vivo.

We then examined MAPK and PI3K/AKT signalling activity in 42 progressing tumours and patient-matched pre-treatment melanoma tissue from 19 patients (GSE65185 and GSE50509^4,16^). Transcriptome analysis was performed using single sample gene set enrichment analysis (ssGSEA)^17^, a non-parametric, unsupervised method that generates an enrichment score representing the degree of absolute enrichment of a gene set for each tumour sample. In 40/42 progressing (PROG) tumours MAPK signalling on therapy was restored to pre-treatment tumour levels (i.e. <10% variation between on-treatment PROG and matched pre-treatment MAPK ssGSEA scores). Importantly, two PROG lesions with no identified MAPK activating mutations, but with acquired PROG-specific PI3K/AKT-activating alterations showed MAPK activity restoration to pre-treatment tumour levels; Pt9-DP1 (PIK3CA^D350G,E545G^;^16^) and Pt13-DP1 (AKT1^Q79K^;^4,5^) (Fig. 1b). We also noted minimal changes in the ssGSEA score of two PI3K/AKT gene-sets on progression, with no evidence that PI3K/AKT activity was significantly changed from pre-therapy to disease progression even in the tumours carrying PI3K/AKT-activating alterations (including NRAS, KRAS mutations) (Fig. 1b).

### PI3K/AKT activity promotes survival, but not proliferation, in response to combination BRAF/MEK inhibition

**Fig. 2:**
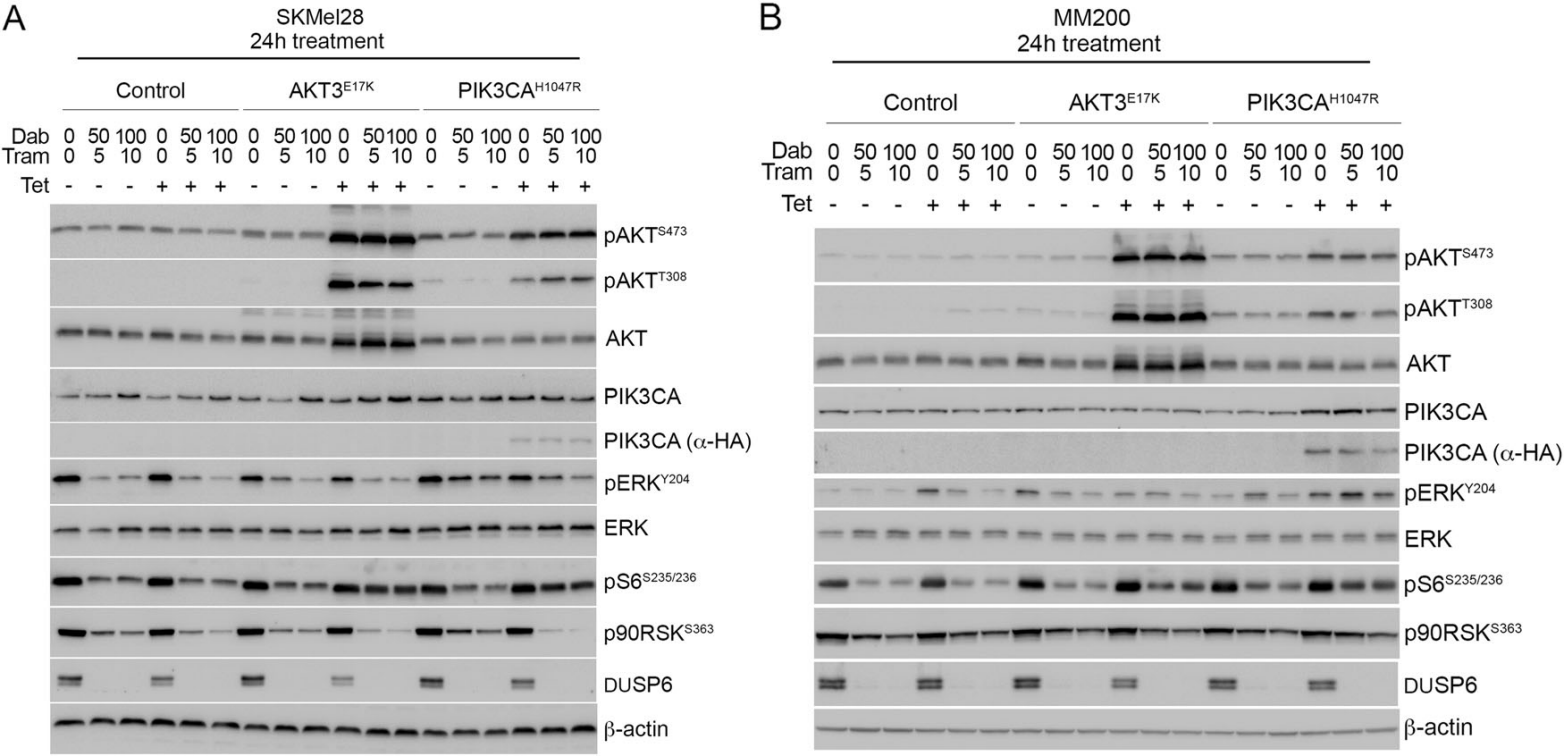
**Induced PI3K/AKT signalling does not influence MAPK activity in response to BRAF/MEK inhibition.** SKMel28 and MM200 melanoma cells expressing tetracycline-inducible expression vector (control), AKT3^E17K^ or PIK3CA^H1047R^ were treated with combination dabrafenib and trametinib for 24 h. Western blot analysis of (**a**) SKMel28 and (**b**) MM200 lysates showing total and phospho-protein markers of MAPK and PI3K/AKT activity. Expression of PIK3CA^H1047R^ is also detected using the HA-epitope tag (α-HA). Dabrafenib and trametinib concentrations shown in nM

To assess the precise contribution of PI3K/AKT activity in BRAF/MEK inhibitor resistance, we selected gain-of-function PIK3CA^H1047R^ and AKT3^E17K^ mutations, both identified in melanoma and associated with BRAF inhibitor resistance^2^. Each activating variant was introduced into the BRAF^V600E^-mutant and BRAF inhibitor-sensitive human melanoma cell lines, MM200 and SKMel28 (Figure S1). Transgene expression was driven by a tetracycline-inducible promoter to regulate mutant protein accumulation, and we achieved near endogenous mutant PIK3CA accumulation, and overexpression of mutant AKT3 in both cell models (Fig. 2). As predicted, tetracycline-induced accumulation of PIK3CA^H1047R^ and AKT3^E17K^ promoted the activation of the PI3K/AKT signalling cascade as noted by the phosphorylation of AKT (i.e. pAKT^S473^ and pAKT^T308^; Fig. 2). PI3K/AKT signalling was also maintained in the presence of combination BRAF/MEK inhibition and this was associated with partial restoration of S6 phosphorylation, which is an effector of both the MAPK and PI3K/AKT cascades (reviewed in ref. ^18^) (Fig. 2). Importantly, MAPK signalling remained inhibited in response to combination BRAF/MEK inhibition regardless of PI3K/AKT activation (i.e. markers of MAPK activity including DUSP6 and p90RSK^S363^ were repressed in the presence of BRAF and MEK inhibitors irrespective of mutant PIK3CA or AKT3 accumulation) (Fig. 2). Although we observed a degree of p-ERK recovery at 24 h post BRAF/MEK inhibition (Fig. 2) we confirmed that phosphorylation of ERK was potently suppressed, in the presence and absence of PI3K/AKT activation, at the earlier time point of 4 h post BRAF/MEK dual inhibition in our transduced cell models (Figure S2A). Further, as previously reported, we noted that p-ERK rebound was sensitive to MEK inhibition^19^, i.e although recovery of p-ERK was detectable at 24 h in the combination BRAF/MEK inhibitor treated parental melanoma cells, rebound was less pronounced in the presence of the MEK inhibitor trametinib (Figure S2B, C).

**Fig. 3:**
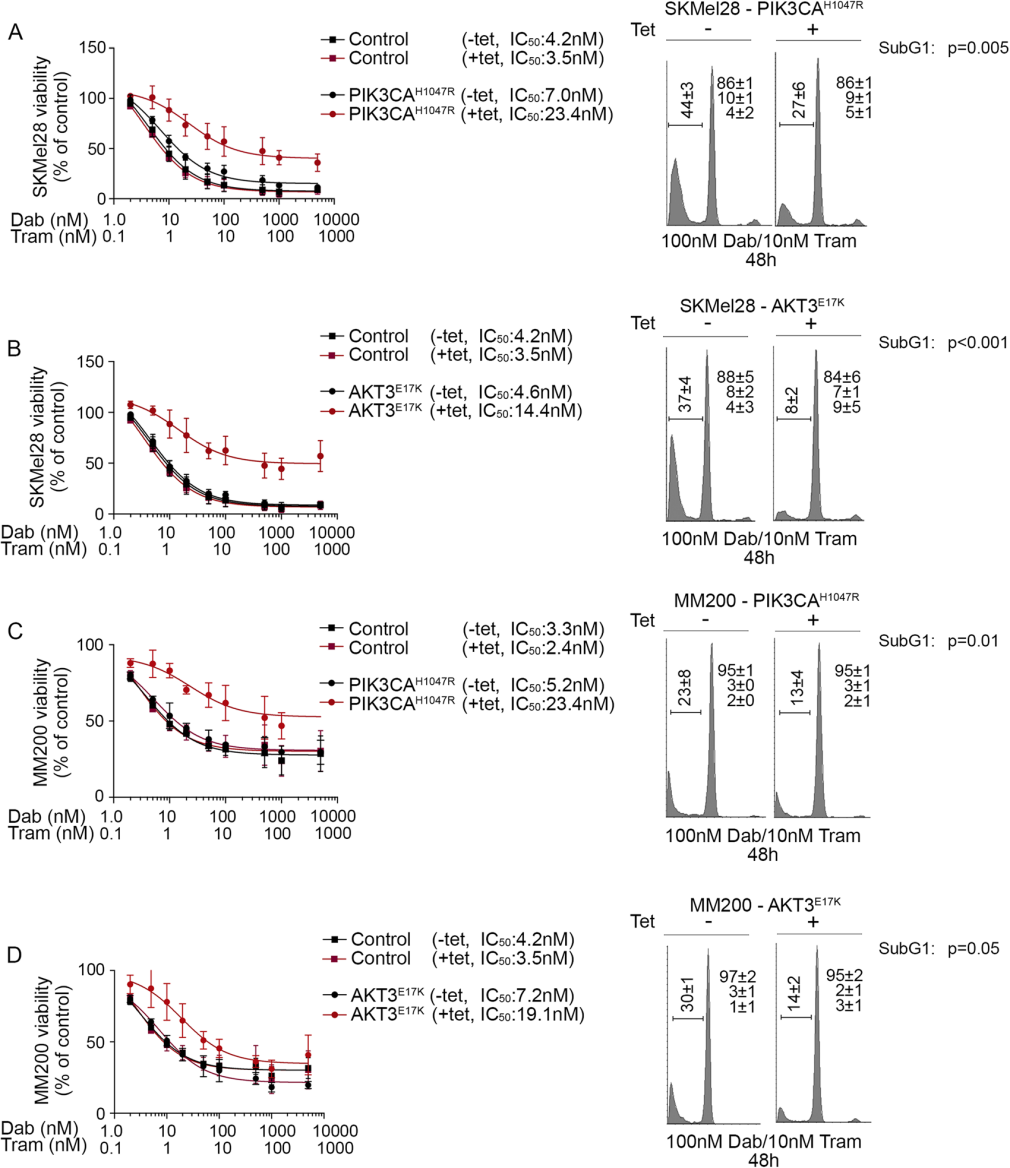
**Short-term PI3K activation promotes cell survival in response to combination BRAF and MEK inhibition.** **a** Viability and cell cycle distribution of SKMel28 cells stably expressing tetracycline-inducible PIK3CA^H1047R^. Viability curves are shown after 72 h of drug treatment and data expressed relative to the DMSO-treated vector controls (mean ± sd). Cell cycle analysis was performed 48 h after treatment with combination dabrafenib (100 nM) and trametinib (10 nM) in the presence or absence of tetracycline. Cell cycle results are the average ± sd of at least three independent experiments. Paired, two-tailed *t*-test was used to compare sub G1 populations in tetracycline-treated and -untreated cells. **b** Viability and cell cycle distribution of SKMel28 cells stably expressing tetracycline-inducible AKT3^E17K^. Assay conditions are as described above. **c** Viability and cell cycle distribution of MM200 cells stably expressing tetracycline-inducible PIK3CA^H1047R^. Assay conditions are as described above. **d** Viability and cell cycle distribution of MM200 cells stably expressing tetracycline-inducible AKT3^E17K^. Assay conditions are as described above

**Fig. 4:**
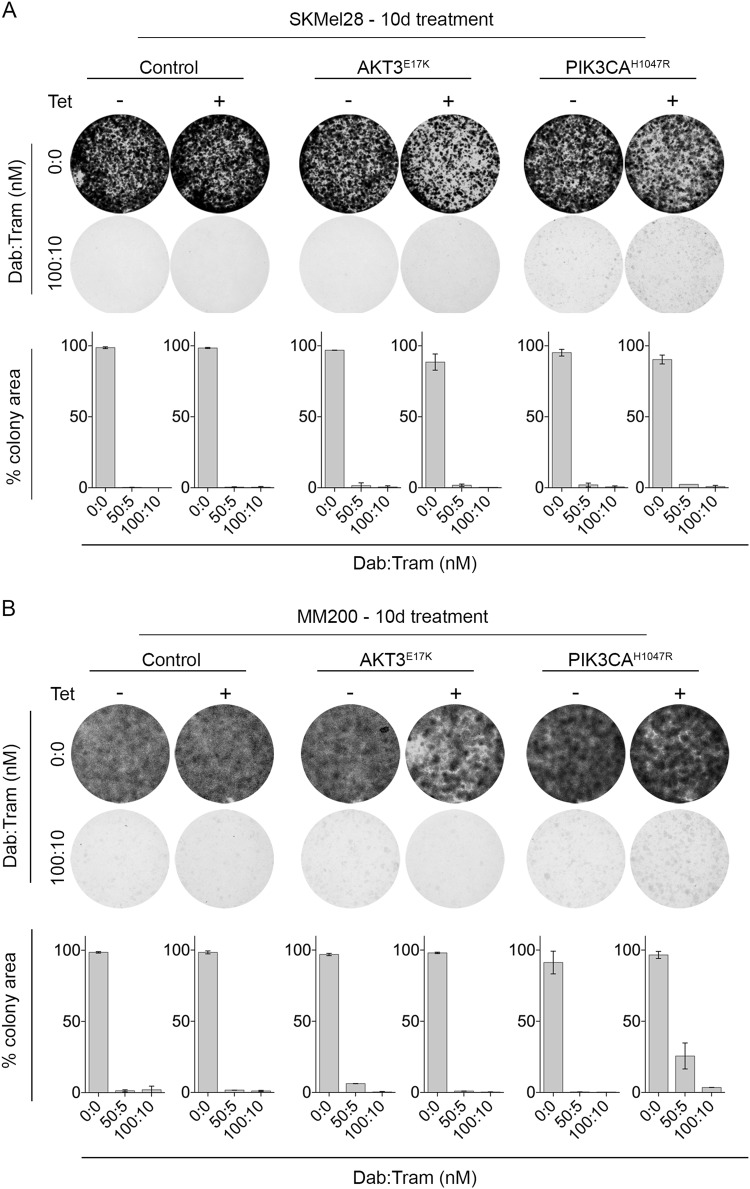
**Long-term PI3K/AKT activation is not sufficient for colony growth in response to combination BRAF and MEK inhibition.** Transduced melanoma cells were seeded at low density and 24 h after seeding were treated with the indicated concentrations of dabrafenib/trametinib every 48–72 h. Colonies were stained with crystal violet 10 days after treatment. Photographs, representative of at least two independent transduction experiments. Histogram, colony formation quantitated using the “Colony Area” ImageJ plugin. Results are the average of at least two independent experiments, each performed in triplicate ± sd. **a** SKMel28 cells stably expressing tetracycline-inducible PIK3CA^H1047R^, AKT3^E17K^ or vector control. **b** MM200 cells stably expressing tetracycline-inducible PIK3CA^H1047R^, AKT3^E17K^ or vector control

To examine the impact of PI3K/AKT signalling on melanoma responses to BRAF/MEK inhibition, MM200 and SKMel28 cells expressing oncogenic PIK3CA or AKT3 mutants were treated with BRAF and MEK inhibitors. The constitutive activation of PI3K/AKT signalling led to diminished BRAF/MEK inhibitor sensitivity in short-term viability assays and reduced the proportion of cells undergoing cell death (Fig. 3 and Figure S3). The survival advantage associated with inducible PIK3CA^H1047R^ expression was also evident in response to single agent BRAF and MEK inhibition (Figure S3). Despite the role of PI3K/AKT signalling in promoting melanoma cell survival, BRAF/MEK inhibitors remained cytostatic in these models and there was no evidence of increased cell proliferation in the presence of PI3K/AKT activation (Fig. 3 and Figure S3). We also confirmed limited outgrowth of colonies in the presence of BRAF/MEK inhibitors in longer-term clonogenic assays (Fig. 4). These data contrast with SKMel28 cells expressing a tetracycline-inducible oncogenic NRAS^Q61K^ mutant; these cells showed resistance in metabolic assays, minimal cell death and long-term colony formation in response to BRAF and MEK inhibition (Figure S4). Critically, whereas MAPK activity was maintained (i.e. expression of DUSP6 and p90RSK^S363^) in the presence of BRAF/MEK inhibition when oncogenic NRAS was expressed (Figure S4), MAPK remained supressed by combination BRAF/MEK inhibitors in the presence of oncogenic PIK3CA and AKT3; i.e. no restoration of the ERK transcription target DUSP6 and lack of ERK-mediated p90RSK phosphorylation (Fig. 2, Figure S2). Moreover, NRAS^Q61K^-driven reactivation of MAPK signalling permitted cell proliferation and colony outgrowth in response to dual BRAF/MEK inhibition (Figure S4), whereas the constitutive activation of PI3K/AKT promoted the survival of cells that were arrested (Fig. 4).

### PI3K/AKT-mediated survival favours the acquisition of MAPK-reactivating alterations and resistance to combination BRAF/MEK inhibition

**Fig. 5:**
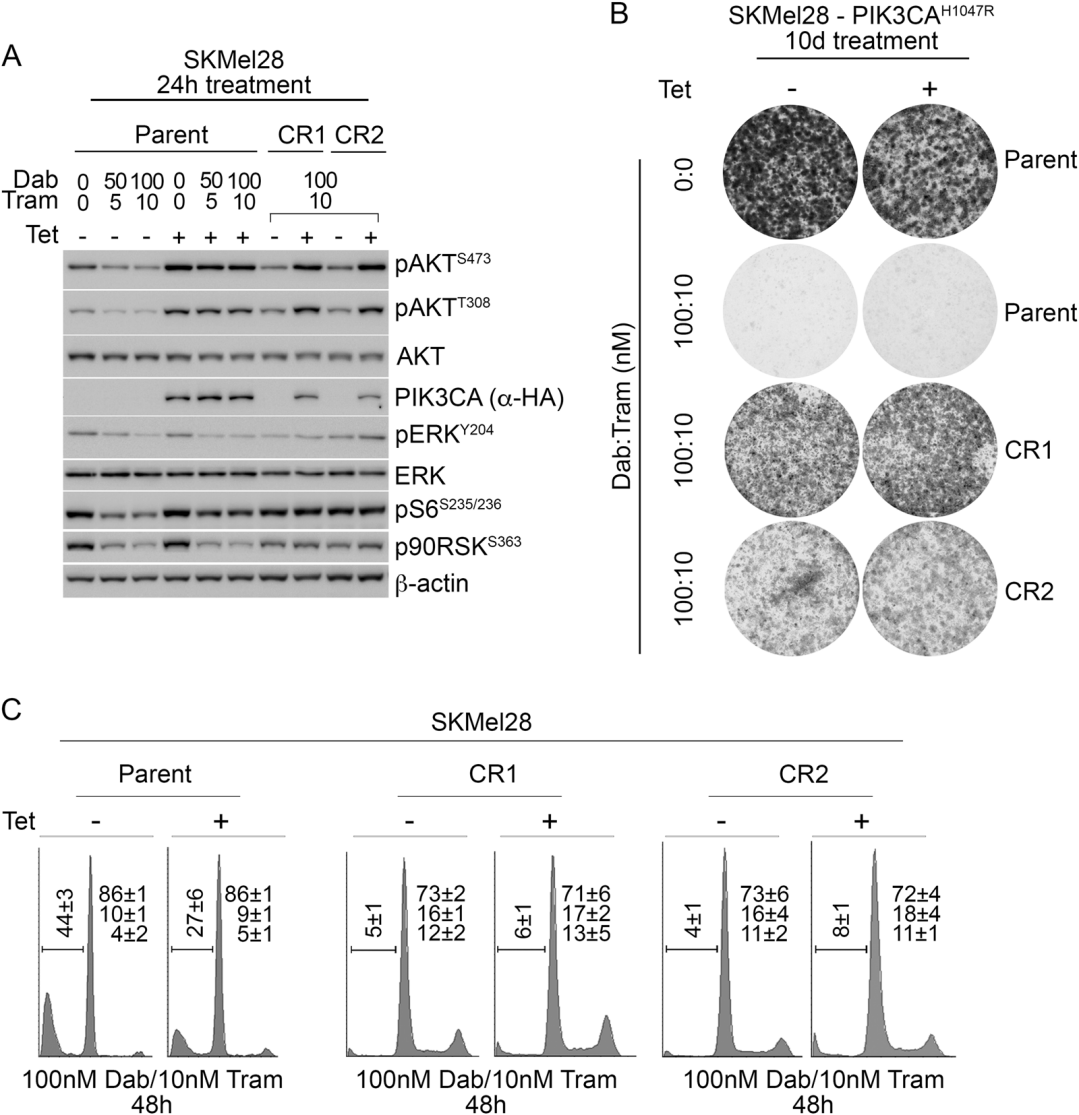
**Acquired resistance to BRAF/MEK inhibition is not dependent on oncogenic PIK3CA signalling and is associated with MAPK reactivation.** SKMel28 melanoma cells transduced with tetracycline-inducible PIK3CA^H1047R^ (Parent) were compared with the CR1 and CR2 resistant sublines, derived after long-term exposure to combination dabrafenib/trametinib. **a** Western blots of lysates showing protein markers of MAPK activity 24 h after treating cells with dabrafenib and trametinib and/or tetracycline to induce PIK3CA^H1047R^ expression. **b** Melanoma cells were seeded at low density and 24 h after seeding were treated with the indicated concentrations of tetracycline and/or dabrafenib/trametinib every 48–72 h. Colonies were stained with crystal violet 10 days after treatment. Photographs, representative of at least two independent transduction experiments. **c** Cell cycle distribution of tetracycline-inducible PIK3CA^H1047R^ (Parent) compared with resistant sublines (CR1, CR2). Cell cycle analysis was performed 48 h after treatment with combination dabrafenib (100 nM) and trametinib (10 nM) in the presence or absence of tetracycline (Tet) (mean ± sd). Results are the average ± sd of at least three independent experiments

To study the dynamics of BRAF/MEK inhibitor resistance in relation to constitutive PI3K/AKT signalling we continuously exposed MM200 and SKMel28 cells to combination dabrafenib and trametinib, in the presence of tetracycline-induced PIK3CA^H1047R^ expression. After 4–6 weeks of continuous BRAF/MEK inhibitor treatment, two independent resistant SKMel28_PIK3CA^H1047R^ sublines (SKMel28 CR1 and CR2) and one MM200_PIK3CA^H1047R^ subline (MM200 CR3) were selected and expanded for analysis. All three CR sublines showed MAPK activity (i.e. phosphorylation of downstream effectors ERK and p90RSK) in the presence of BRAF/MEK inhibition, and importantly MAPK signalling did not require tetracycline-induced PIK3CA^H1047R^ expression (Fig. 5). This contrasted with the parental transduced SKMel28 and MM200 cell models that were not continuously exposed to BRAF/MEK inhibitors. These cells responded to BRAF/MEK inhibition with suppression of MAPK signalling in the presence of induced mutant PIK3CA and AKT3 (Fig. 2). Cell cycle and clonogenic analyses of the CR sublines confirmed that they continued proliferating in the presence of BRAF/MEK inhibitors, and proliferation was independent of the tetracycline-induced PIK3CA^H1047R^ expression (Fig. 5 and S5).

**Fig. 6:**
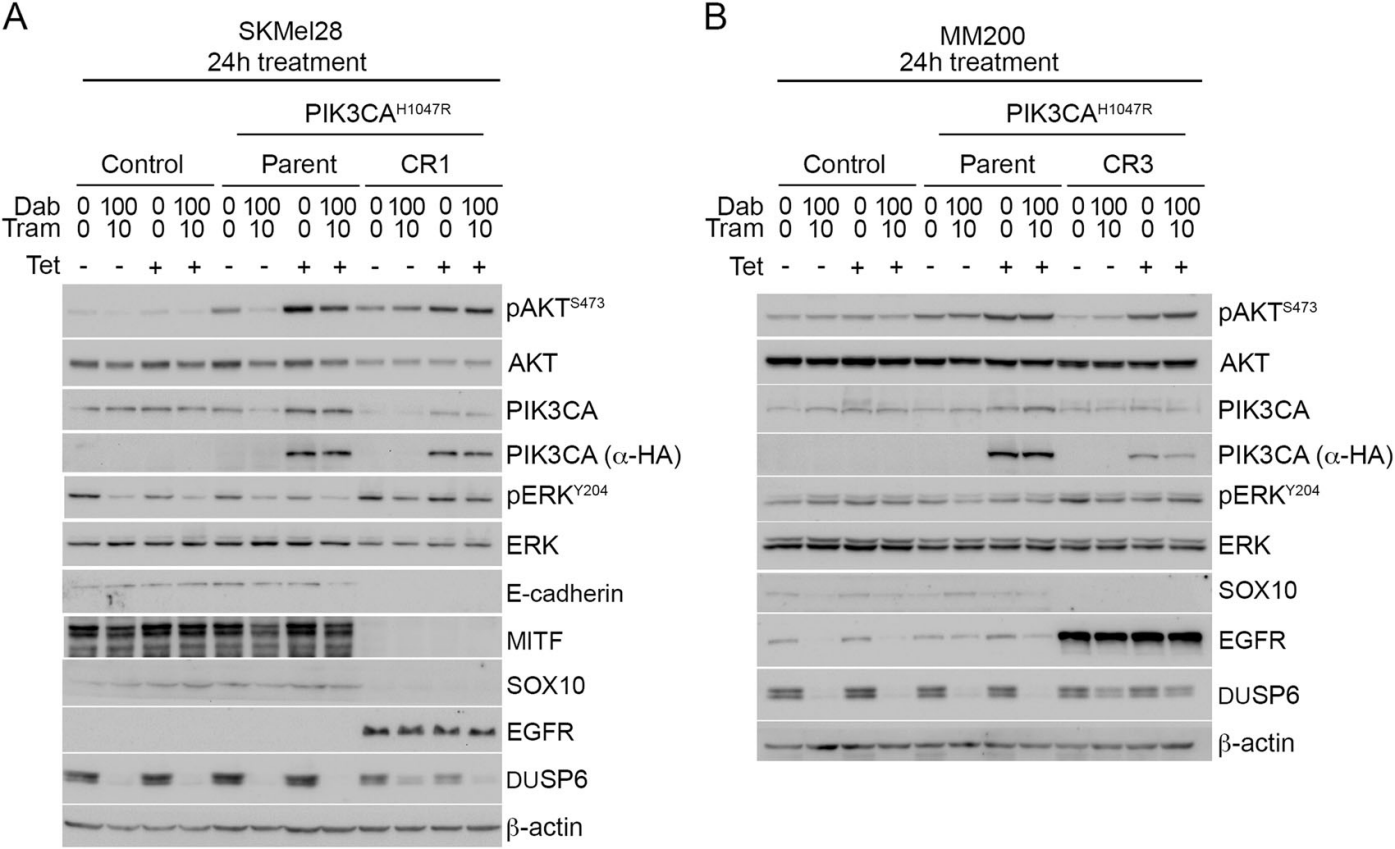
**Acquired resistance to BRAF/MEK inhibition associated with de-differentiated phenotype, and not dependent on PI3K or AKT3 transgene expression.** Melanoma cells transduced with vector (Control), tetracycline-inducible PIK3CA^H1047R^ (Parent) were compared with the resistant sublines (CR1, CR3), derived after long-term exposure to combination dabrafenib/trametinib. Western blots of lysates showing protein markers of differentiation (MITF, SOX10), MAPK and PI3K/AKT signalling 24 h after treating (**a**) SKMel28 or (**b**) MM200 cells with dabrafenib (Dab) and trametinib (Tram) and/or tetracycline (Tet) to induce PIK3CA^H1047R^ expression

We investigated potential resistance mechanisms in the SKMel28 CR1 and MM200 CR3 sublines and they were both found to display features of the de-differentiated resistance phenotype, including the upregulation of the receptor tyrosine kinase EGFR, loss of MITF in the SKMel28 cells, and diminished expression of transcriptional regulator SOX10 ^20–22^. Loss of the epithelial marker E-cadherin was also observed in SKMel28 CR1 (E-cadherin and MITF were not detectable in the MM200 parental cell line) (Fig. 6). These markers of de-differentiation and PI3K/AKT activation (i.e. pAKT^Ser473^) were evident in the presence and absence of PIK3CA^H1047R^ expression. These data confirm that resistance mechanisms leading to the re-activation of MAPK signalling were acquired in the SKMel28 and MM200 CR sublines. Consistent with previous reports, the de-differentiated CR1 and CR3 clones were highly drug resistant (in the absence of PIK3CA^H1047R^ induction) and showed limited cell death even when both the PI3K/AKT and MAPK cascades were inhibited^21,23^. The combination of the dual PI3K/mTOR inhibitor BEZ235 and the ERK inhibitor SCH772984, potently inhibited MAPK and PI3K activity, induced a degree cell death in both CR1 (Figure S6) and CR3 sublines (data not shown). In contrast, the inhibition of MAPK or PI3K/AKT signalling alone, via ERK inhibition or PI3K/mTOR inhibition, did not result in the induction of cell death in either the CR1 (Figure S6) and CR3 (data not shown) clones. Thus, these resistant sublines utilise multiple signalling cascades, including the MAPK and PI3K/AKT pathways as redundant survival pathways, and this signalling reorganisation was no longer dependent on oncogenic PIK3CA^H1047R^.

## Discussion

The majority of patients with metastatic BRAF^V600E/K^-mutant melanoma will fail BRAF inhibitor-based targeted therapy within the first year of treatment. In almost all resistant tumours the MAPK pathway is reactivated either via activating mutations or amplification in BRAF, MEK1/2 or NRAS or via the transcriptomic upregulation of receptor tyrosine kinases, including PDGFRß and c-MET^2,16^. Two secondary resistance pathways have also been identified as less common resistance effectors; the PI3K/AKT signalling cascade and the ‘MITF-low’ de-differentiation transcription programme^21^. Intriguingly, whereas mutations in BRAF, NRAS and MEK1/2 usually occur in isolation within resistant tumours, non-MAPK alterations often co-exist with other genetic changes^2^, and this suggests that genetic and transcriptomic changes acquired during treatment failure may co-operate to select and promote resistance. In EGFR-mutant non-small cell lung cancer cell models, for instance, the sequential activation of PI3K/AKT and MAPK signalling co-operatively produced highly proliferative cells that survived exposure to EGFR inhibition^24^. Similarly, PTEN loss, which promotes an adaptive rebound in AKT activity in response to BRAF inhibition^25^, often co-exists with MAPK-activating mutations in BRAF inhibitor resistant melanomas^2^.

In this study, we investigated the precise contribution of PI3K/AKT signalling in melanoma resistance to BRAF/MEK inhibition. Mutations activating this pathway are not common in vivo and have never been reported to occur in isolation or in the absence of MAPK signalling in melanoma. In addition, studies on melanoma cell lines indicated that the impact of activating AKT mutations was a cell context dependent response^25^. The AKT^Q79K^ mutation, for instance, was identified in a single BRAF inhibitor-resistant melanoma and was shown to selectively promote BRAF inhibitor resistance in PTEN-wild type melanoma cells that display weak AKT phosphorylation in response to BRAF inhibition^25^. Interestingly, the SKMel28 or MM200 melanoma cells used in this work, are PTEN wild type and did not show AKT^T308^ phosphorylation in response to BRAF/MEK inhibition. Thus, we expected PI3K/AKT signalling would confer robust resistance to BRAF/MEK inhibition in both these melanoma cell models. We also confirmed that the PROG melanomas carrying the AKT^Q79K^ or PIK3CA^D350G/E545G^ mutations both displayed re-activation of MAPK activity, suggesting that PI3K/AKT activation may have contributed to the selection of additional events that conferred robust BRAF/MEK inhibitor resistance.

Evaluation of clinical data also suggests that PI3K/AKT activation is not sufficient to confer BRAF and MEK inhibitor resistance in melanoma. Partial responses are seen in patients with pre-treatment PTEN tumour loss^7,9^, with pre-treatment AKT phosphorylation, and with pre-treatment AKT3 and RAC1 activating mutations^3,7^. The fact that PI3K/AKT activating mutations commonly co-occur with other genetic alterations^2^, and that new MAPK-reactivating mutations are acquired in resistant tumours with pre-existing PI3K/AKT activating mutations indicate that PI3K/AKT signalling favours the selection and expansion of resistant tumour subclones. These data are in line with the observations that PTEN-loss is only weakly associated with shorter progression free survival to BRAF inhibition^9^.

Our data confirm that PI3K/AKT activation, via gain of function AKT or PIK3CA mutations, promotes the survival, but not proliferative escape, of melanoma cells exposed to dual BRAF/MEK inhibition. The survival of arrested cells allows for the selection of resistant subclones with additional acquired changes that re-activate MAPK signalling and promote proliferation in the presence of MAPK inhibitors. In our resistant cell models, combination BRAF/MEK inhibitor escape was associated with the dedifferentiated ‘MITF-low’ programme, a programme that confers resistance to BRAF, MEK and ERK inhibition and also to the combination of BRAF/MEK inhibitors^21^. The resistant sublines showed activation of PI3K/AKT and MAPK signalling that were independent of mutant BRAF, mutant AKT and mutant PIK3CA, but remained partially sensitive to downstream inhibition of both pathways. Myristoylated AKT3 has also been shown to partially protect melanoma cells from BRAF-inhibitor induced apoptosis without promoting MAPK activity^26^. Thus, during this multi-step model of BRAF/MEK inhibitor resistance the initial effectors of tumour cell survival may no longer be required after the evolution of durable drug resistance and this has important clinical implications. First, initial treatment needs to target not only the oncogenic drivers but also potential modulators of response, such as PI3K/AKT signalling. There are no clinical trials that have addressed combined MAPK/PI3K inhibition in the context of delayed resistance. Second, the sequential or concurrent inhibition of the initial drivers is unlikely to be effective once high-level RAF inhibitor resistance develops. Third, paracrine stimulation of response modulators, including the stimulation of tumoural PI3K/AKT activity via stroma-derived secreted factors (i.e. hepatocyte growth factor, epidermal growth factor)^27^ need to be considered when designing upfront combination therapies.

## Methods and methods

### Immunoblotting

Total cellular proteins were extracted at 4 °C using RIPA lysis buffer containing protease inhibitors and phosphatase inhibitors (Roche, Basel, Switzerland). Proteins (20 µg) were resolved on 8–12% SDS-polyacrylamide gels and transferred to Immobilon-P membranes (Millipore, Bedford, MA). Western blots were probed with antibodies against p-ERK (Tyr204, 1:2000; E4; Santa Cruz, CA), total ERK (1:2000; 137F5; Cell Signalling, Danvers, MA), p-AKT (Ser473, 1:1000, 736E11, Cell Signalling), total AKT (1:1000; 40D4, Cell Signalling), p-S6 (Ser235/236, 1:2000, 2F9, Cell Signalling), p-RSK1/2/4 (Ser363, 1:2000, Santa Cruz), total PI3K (1:1000, C73F8, Cell Signalling), HA-tag (1:1000, 6E2, Cell Signalling), DUSP6 (1:1000, EPR129Y, Abcam), EGF receptor (1:1000, D38B1, Cell Signalling), p-EGF receptor (Tyr1068, 1:500, Cell Signalling), MITF (1:1000, C5, Calbiochem), SOX10 (1:500, N-20, Santa Cruz), E-cadherin (1:1000, SHE78-7, Invitrogen), NRas (1:1000, F155, Santa Cruz) and ß-Actin (1:3000, I-19, Santa Cruz). Each western blot was performed using at least two biological replicates. For cell transduction work we performed two biological replicates from each of two independent transductions.

### Pharmacological and clonogenic growth assays

Pharmacological growth inhibition assays were performed in 96 well plates using 2 × 10^3^ cells per well, seeded in the presence of absence of 0.5 µg/ml tetracycline. 24 h after seeding, cells were treated with serial dilutions of dabrafenib and trametinib in a 10:1 concentration ratio. Cells were incubated for an additional 72 h and cell viability was measured using the Cell Proliferation Aqueous MTS assay (Promega) according to the manufacturer’s protocol. Absorbance was quantitated on a Pherastar FS plate reader at a wavelength of 490 nm. MTS assays were performed at least twice each in triplicate, with independent transductions. Clonogenic assays were performed as previously described^4^. Quantitative analysis of colony formation was performed using the ImageJ plugin ColonyArea^28^.

### Cell cycle and apoptosis analysis

Adherent and floating cells were combined and cell cycle and apoptosis analyses were performed as previously described^29^. 100 nM dabrafenib/10 nM trametinib concentrations reflect 5–10x the IC_50_ values derived from a panel of sensitive melanoma cell lines^3^. Cell cycle analyses were performed using at least three biological replicates, and data presented as mean ± sd.

### Cell culture, constructs and lentivirus transductions

SKMel28 and MM200 melanoma cells were kindly provided by P. Hersey, University of Sydney, and grown in Dulbecco’s Modified Eagle Medium (DMEM) with 10% FBS and glutamine (Sigma-Aldrich) and cultured in a 37 °C incubator with 5% CO_2_. Cell authentication was confirmed using the StemElite ID system from Promega and all cells tested negative for mycoplasma (MycoAlert Mycoplasma Detection Kit, Lonza, Basel). Lentiviruses were produced in HEK293T cells as described previously^30^. Cells were infected using a multiplicity of infection of 1–5 to provide an efficiency of infection above 90%. Cells transduced to express the tetracycline receptor from the *pLenti3.3/TR* vector were used in all transduction experiments. The MYC-tagged AKT3^E17K^, HA-tagged PIK3CA^H1047R^ or MYC-tagged NRAS^Q61K^ were each cloned into the *plenti6.3/T0/V5-DEST* lentiviral vector (Thermo Fisher). Cells were selected with 500 µg/ml geneticin (G418 Sulphate) and 4 µg/ml blasticidin (Life Technologies) to ensure maintenance of transgene expression.

### Gene expression analysis

Transcriptome analysis of BRAF and MEK inhibitor treated melanoma patient samples (GSE65185 and GSE50509) was performed using single sample gene set enrichment analysis (ssGSEA)^17^. ssGSEA is non-parametric, unsupervised method of gene set enrichment that generates an enrichment score representing the degree of absolute enrichment of a gene set for each tumour sample. The gene sets used in ssGSEA analysis consisted of the Hallmark gene set collection, a refined collection of gene signatures that was derived from multiple founder gene sets to define specific biological processes^31^. The Hallmark PI3K_AKT_mTOR and MTORC1_signalling gene sets include 105 and 200 genes, respectively to measure mTORC1 signalling and PI3K signalling via AKT and mTORC1^31^. We also examined a MAPK activation gene set which consists of 48 MEK-dependent genes derived from a panel of BRAF^V600E^-mutant melanoma cell lines^32^. This MAPK gene set accurately reflected MAPK inhibition early on treatment patient-derived melanoma samples^3^.

### Statistical analysis

Statistical analysis was performed using Prism 7.0 for Mac OS X (GraphPad Software). Differences between two groups were calculated using paired, two-sided *t*-test or Mann–Whitney test, as indicated. In all cases, *p*-values of <0.05 were considered to be significant. The Brown-Forsythe test was used to test for equal variance in unpaired tests.

## Electronic supplementary material

Supplementary Tables and Figure legends

Figure S1

Figure S2

Figure S3

Figure S4

Figure S5

Figure S6

## References

[1] Long GV (2016). Overall survival and durable responses in patients with braf v600-mutant metastatic melanoma receiving dabrafenib combined with trametinib. J. Clin. Oncol..

[2] Johnson DB (2015). Acquired BRAF inhibitor resistance: a multicenter meta-analysis of the spectrum and frequencies, clinical behaviour, and phenotypic associations of resistance mechanisms. Eur. J. Cancer.

[3] Long GV (2014). Increased MAPK reactivation in early resistance to dabrafenib/trametinib combination therapy of BRAF-mutant metastatic melanoma. Nat. Commun..

[4] Rizos H (2014). BRAF inhibitor resistance mechanisms in metastatic melanoma: spectrum and clinical impact. Clin. Cancer Res..

[5] Shi H (2014). Acquired resistance and clonal evolution in melanoma during BRAF inhibitor therapy. Cancer Discov..

[6] Wagle N (2011). Dissecting therapeutic resistance to RAF inhibition in melanoma by tumor genomic profiling. J. Clin. Oncol..

[7] Trunzer K (2013). Pharmacodynamic effects and mechanisms of resistance to vemurafenib in patients with metastatic melanoma. J. Clin. Oncol..

[8] Meyer S (2012). A seven-marker signature and clinical outcome in malignant melanoma: a large-scale tissue-microarray study with two independent patient cohorts. PLoS ONE.

[9] Nathanson KL (2013). Tumor genetic analyses of patients with metastatic melanoma treated with the BRAF inhibitor dabrafenib (GSK2118436). Clin. Cancer Res..

[10] Catalanotti F. et al. PTEN Loss-of-Function Alterations Are Associated With Intrinsic Resistance to BRAF Inhibitors in Metastatic Melanoma. *Precis Oncol*. **1**, (2017). 10.1200/PO.16.00054 PMC7446400 32913971

[11] Nissan MH (2014). Loss of NF1 in cutaneous melanoma is associated with RAS activation and MEK dependence. Cancer Res..

[12] Xing F (2012). Concurrent loss of the PTEN and RB1 tumor suppressors attenuates RAF dependence in melanomas harboring (V600E)BRAF. Oncogene.

[13] Ascierto PA (2013). MEK162 for patients with advanced melanoma harbouring NRAS or Val600 BRAF mutations: a non-randomised, open-label phase 2 study. Lancet Oncol..

[14] Won JK (2012). The crossregulation between ERK and PI3K signaling pathways determines the tumoricidal efficacy of MEK inhibitor. J. Mol. Cell Biol..

[15] Castellano E, Downward J (2011). RAS interaction with PI3K: more than just another effector pathway. Genes Cancer.

[16] Hugo W (2015). Non-genomic and immune evolution of melanoma acquiring MAPKi resistance. Cell.

[17] Barbie DA (2009). Systematic RNA interference reveals that oncogenic KRAS-driven cancers require TBK1. Nature.

[18] Mendoza MC, Er EE, Blenis J (2011). The Ras-ERK and PI3K-mTOR pathways: cross-talk and compensation. Trends Biochem. Sci..

[19] Paraiso KH (2010). Recovery of phospho-ERK activity allows melanoma cells to escape from BRAF inhibitor therapy. Br. J. Cancer.

[20] Konieczkowski DJ (2014). A melanoma cell state distinction influences sensitivity to MAPK pathway inhibitors. Cancer Discov..

[21] Muller J (2014). Low MITF/AXL ratio predicts early resistance to multiple targeted drugs in melanoma. Nat. Commun..

[22] Riesenberg S (2015). MITF and c-Jun antagonism interconnects melanoma dedifferentiation with pro-inflammatory cytokine responsiveness and myeloid cell recruitment. Nat. Commun..

[23] Carlino MS (2014). Differential activity of MEK and ERK inhibitors in BRAF inhibitor resistant melanoma. Mol. Oncol..

[24] Cortot AB (2013). Resistance to irreversible EGF receptor tyrosine kinase inhibitors through a multistep mechanism involving the IGF1R pathway. Cancer Res..

[25] Shi H (2014). A novel AKT1 mutant amplifies an adaptive melanoma response to BRAF inhibition. Cancer Discov..

[26] Shao Y, Aplin AE (2010). Akt3-mediated resistance to apoptosis in B-RAF-targeted melanoma cells. Cancer Res..

[27] Straussman R (2012). Tumour micro-environment elicits innate resistance to RAF inhibitors through HGF secretion. Nature.

[28] Guzman C, Bagga M, Kaur A, Westermarck J, Abankwa D (2014). ColonyArea: an ImageJ plugin to automatically quantify colony formation in clonogenic assays. PLoS ONE.

[29] Gallagher S, Kefford RF, Rizos H (2005). Enforced expression of p14ARF induces p53-dependent cell cycle arrest but not apoptosis. Cell Cycle.

[30] Haferkamp S (2009). Oncogene-induced senescence does not require thep16(INK4a) or p14ARF melanoma tumor suppressors. J. Invest. Dermatol..

[31] Liberzon A (2015). The Molecular Signatures Database (MSigDB) hallmark gene set collection. Cell Syst..

[32] Pratilas CA (2009). V600E)BRAF is associated with disabled feedback inhibition of RAF-MEK signaling and elevated transcriptional output of the pathway. Proc. Natl Acad. Sci. USA.

[33] Moriceau G (2015). Tunable-combinatorial mechanisms of acquired resistance limit the efficacy of BRAF/MEK cotargeting but result in melanoma drug addiction. Cancer Cell..

