# Supplementary material for: Oncogenic PI3K/AKT promotes the step-wise evolution of combination BRAF/MEK inhibitor resistance in melanoma
Source: Oncogenesis. 2018 Sep 20;7(9):72. doi: 10.1038/s41389-018-0081-3 (PMC6148266; doi:10.1038/s41389-018-0081-3)
Supplement: Supplementary file 1 — Supplementary Tables and Figure legends [file 41389_2018_81_MOESM1_ESM.docx]

**Table S1** Correlation of BRAF and MEK inhibitor sensitivity data and cancer oncogenic alterations

|  | **BRAF inhibitor**  **(dabrafenib)** | | | **MEK inhibitor (trametinib)** | | | **MEK inhibitor**  **(CI-1040)** | | |
| --- | --- | --- | --- | --- | --- | --- | --- | --- | --- |
| *Genotype* | *p-value* | *%FDR* | *No. cell lines* | *p-value* | *%FDR* | *No. cell lines* | *p-value* | *%FDR* | *No. cell lines* |
| *BRAF* mutant | 3.0e-44 | <0.001 | 72 | 4.3e-05 | 0.766 | 77 | 1.8e-05 | 0.269 | 70 |
| *NRAS* mutant | 0.157 | 84.7 | 54 | 6.2e-07 | 0.335 | 55 | 1.0e-05 | 0.236 | 56 |
| *KRAS* mutant | 0.19 | 84.7 | 112 | 5.3e-06 | 0.144 | 112 | 3.5e-06 | 0.159 | 109 |

FDR, false discovery rate; p-value from ANOVA of drug-gene interaction. Data derived from the Genomics of Drug Sensitivity in Cancer database^3^

**Table S2** PI3K/AKT and MAPK activating mutations in matched PRE and PROG melanoma biopsies

| **Patient** | **PRE tumour mutations^1^** | **PROG tumour mutations^2^** | **Treatment** | **Reference** |
| --- | --- | --- | --- | --- |
| C5 | AKT3^E17K^, RAC1^P29S^ | AKT3^E17K^, RAC1^P29S^, MEK2^E207K^, NRAS^G12D^ | CombiDT | ^13^ |
| C6 | PTEN^P169S,K164fs,^ RAC1^P29L^ | PTEN^P169S,K164fs^, RAC1^P29L^, NRAS^Q61K^ | CombiDT | ^13^ |
| C3 | PTEN^I33del^ | PTEN^I33del^, MEK2^C125S^ | CombiDT | ^13^ |
| 60 | PIK3CA^L540F^ | PIK3CA^L540F^, STAG3^Ser1016fs^ | BRAFi | ^24, 29^ |
| 58 | PTEN^K128T^ | PTEN^K128T^, MITF amplification | BRAFi | ^29^ |

^1^Mutations activating PI3K/AKT pathway in pre-treatment (PRE) tumours are shown

^2^Mutations acquired during BRAF and/or MEK inhibitor progression (PROG tumours) are shown

CombiDT, combination dabrafenib plus trametinib; BRAFi, BRAF inhibitor monotherapy

**Figure S1 Response of SKMel28 and MM200 cells to BRAF and/or MEK inhibition**

Cell cycle distribution of SKMel28 and MM200 cells treated with either DMSO (Control), 100nM dabrafenib (Dab) alone, 10nM trametinib (Tram) alone or combination 100nM dabrafenib and 10nM trametinib for 48 h.

**Figure S2 Induced PIK3CA^H1047R^ does not influence MAPK activity in response to BRAF/MEK inhibition**

1. SKMel28 and MM200 melanoma cells expressing tetracycline-inducible expression vector (Control) or PIK3CA^H1047R^ were treated with combination dabrafenib and trametinib as indicated for 4h. Western blot analysis of lysates showing total and phospho-protein markers of MAPK and PI3K/AKT activity. Dabrafenib and trametinib concentrations shown in nM.
2. Response of SKMel28 parental cells to dabrafenib, trametinib or combination dabrafenib and trametinib over a 24h time course. Western blot analysis showing total and phospho-protein markers of MAPK activity.
3. Response of MM200 parental cells to dabrafenib, trametinib or combination dabrafenib and trametinib over a 24h time course. Western blot analysis showing total and phospho-protein markers of MAPK activity.

**Figure S3 Effect of short-term PIK3CA^H1047R^ induction on melanoma cell responses to combination BRAF/MEK inhibition**

1. Cell cycle distribution data of SKMel28 cells stably expressing tetracycline-inducible PIK3CA^H1047R^ treated with DMSO or the indicated doses of dabrafenib and/or trametinib for 48 hours. Results are the average ± sd of at least three independent experiments.
2. Cell cycle distribution data of MM200 cells stably expressing tetracycline-inducible PIK3CA^H1047R^. Assay conditions are as described above.
3. Cell cycle distribution data of SKMel28 cells stably expressing tetracycline-inducible AKT3^E17K^. Assay conditions are as described above.
4. Cell cycle distribution data of MM200 cells stably expressing tetracycline-inducible AKT3^E17K^. Assay conditions are as described above.

**Figure S4 NRAS^Q61K^-driven resistance to combination BRAF and MEK inhibition**

SKMel28 melanoma cells were transduced with and selected for tetracycline-inducible NRAS^Q61K^

1. Western blots of lysates showing protein markers of MAPK activity 24h after treating cells with dabrafenib and trametinib and/or tetracycline to induce NRAS^Q61K^ expression.
2. Viability of SKMel28 cells stably expressing tetracycline-inducible NRAS^Q61K^. Viability curves are shown after 72h of drug treatment and data expressed relative to the DMSO-treated controls (mean ± sd).
3. Cell cycle analysis of SKMel28 cells stably expressing tetracycline-inducible NRAS^Q61K^ was performed 48 hours after treatment with combination dabrafenib (100nM) and trametinib (10nM) in the presence or absence of tetracycline (Tet). Paired, two-tailed t-test was used to compare sub G1 populations in tetracycline-treated and -untreated cells. Results are the average ± sd of at least three independent experiments.
4. SKMel28 melanoma cells were seeded at low density and 24 hours after seeding were treated with the indicated concentrations dabrafenib/trametinib in the presence or absence of tetracycline (Tet). Colonies were stained with crystal violet 10 days after treatment Photographs, representative of at least two independent transduction experiments.

**Figure S5 Acquired resistance to BRAF/MEK inhibition is not dependent on oncogenic PIK3CA^H1047R^ and was associated with MAPK reactivation**

MM200 melanoma cells transduced with tetracycline-inducible PIK3CA^H1047R^ (Parent) were compared with the CR3 resistant sublines, derived after long-term exposure to combination dabrafenib/trametinib.

1. Western blots of lysates showing protein markers of MAPK activity 24h after treating cells with dabrafenib and trametinib and/or tetracycline to induce PIK3CA^H1047R^ expression.
2. Melanoma cells were seeded at low density and 24 hours after seeding were treated with the indicated concentrations of tetracycline and/or dabrafenib/trametinib every 48 to 72 hours. Colonies were stained with crystal violet 10 days after treatment. Photographs, representative of at least two independent transduction experiments.
3. Cell cycle distribution of tetracycline-inducible PIK3CA^H1047R^ (Parent) compared with the resistant subline (CR3). Cell cycle analysis was performed 48 hours after treatment with combination dabrafenib and trametinib in the presence or absence of tetracycline. Results are the average ± sd of at least three independent experiments.

**Figure S6 Dual inhibition of the MAPK and PI3K pathways is required to inhibit proliferative and survival pathways in de-differentiated melanoma**

SKMel28 cells transduced with tetracycline-inducible vector (Control) and CR1 resistant sublines derived after long-term exposure to combination dabrafenib/trametinib, were treated with combinations of 100nM dabrafenib/10nM trametinib (D/T); 2µM PI3K/mTOR inhibitor BEZ235 (BEZ) 500nM ERK inhibitor SCH772984 (SCH). Tetracycline (Tet) was also used, as indicated to induce ectopic PIK3CA^H1047R^ expression.

1. Western blots of lysates showing protein markers of MAPK and PI3K/AKT activity 24h after treating cells with the indicated inhibitors.
2. SKMel28 CR1 cells were treated with DMSO (Control) or indicated inhibitors and percentage of sub-G1 cells (mean ± sd) determined by FACS analysis 48h after treatment with DMSO (Control) or the indicated inhibitors. Results are the average ± sd of at least three independent experiments.
