# Supplementary figures and images for: Oncogenic PI3K/AKT promotes the step-wise evolution of combination BRAF/MEK inhibitor resistance in melanoma
Source: Oncogenesis. 2018 Sep 20;7(9):72. doi: 10.1038/s41389-018-0081-3 (PMC6148266; doi:10.1038/s41389-018-0081-3)

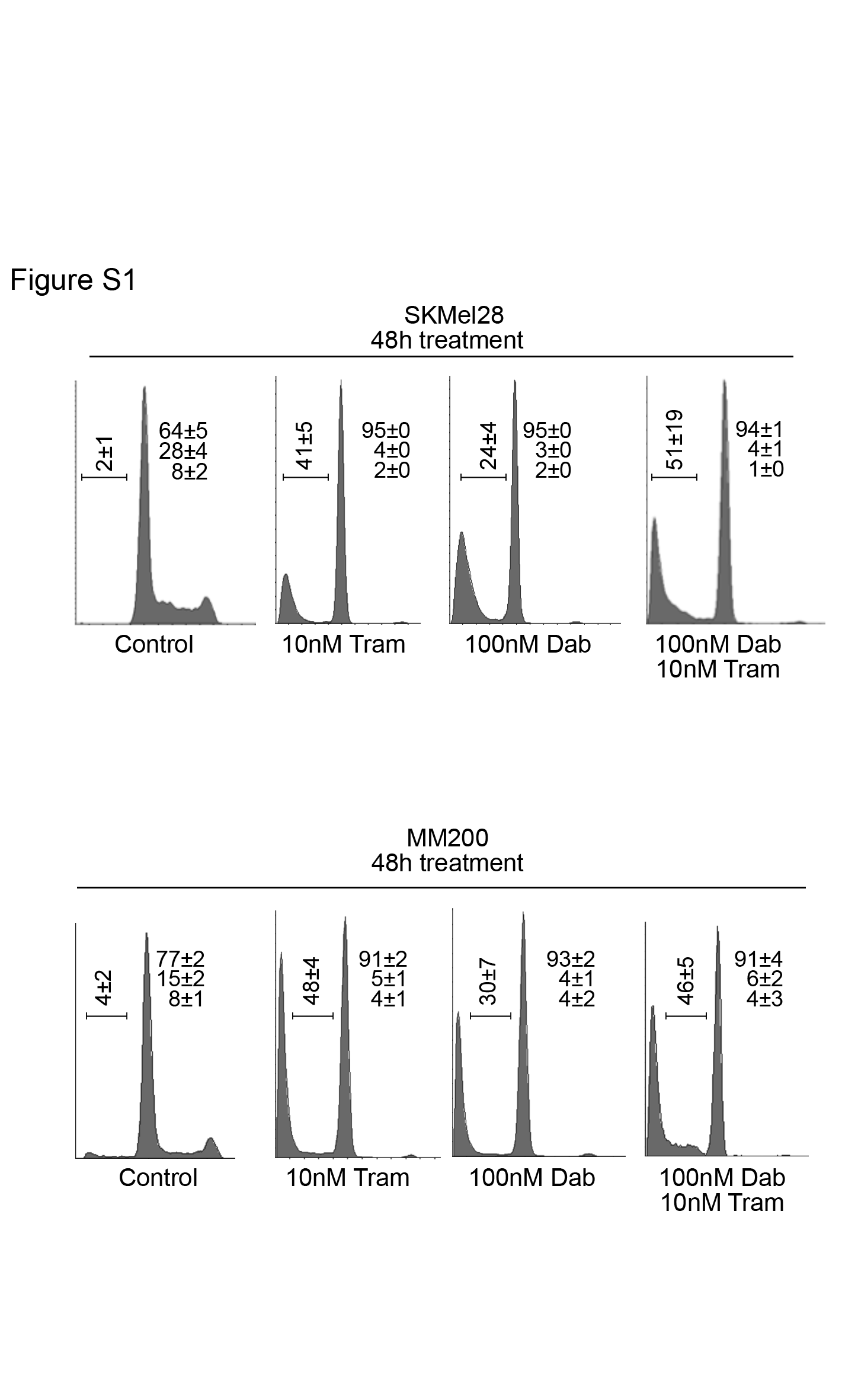

Supplement: Supplementary file 2 — Figure S1 [file 41389_2018_81_MOESM2_ESM.tif]

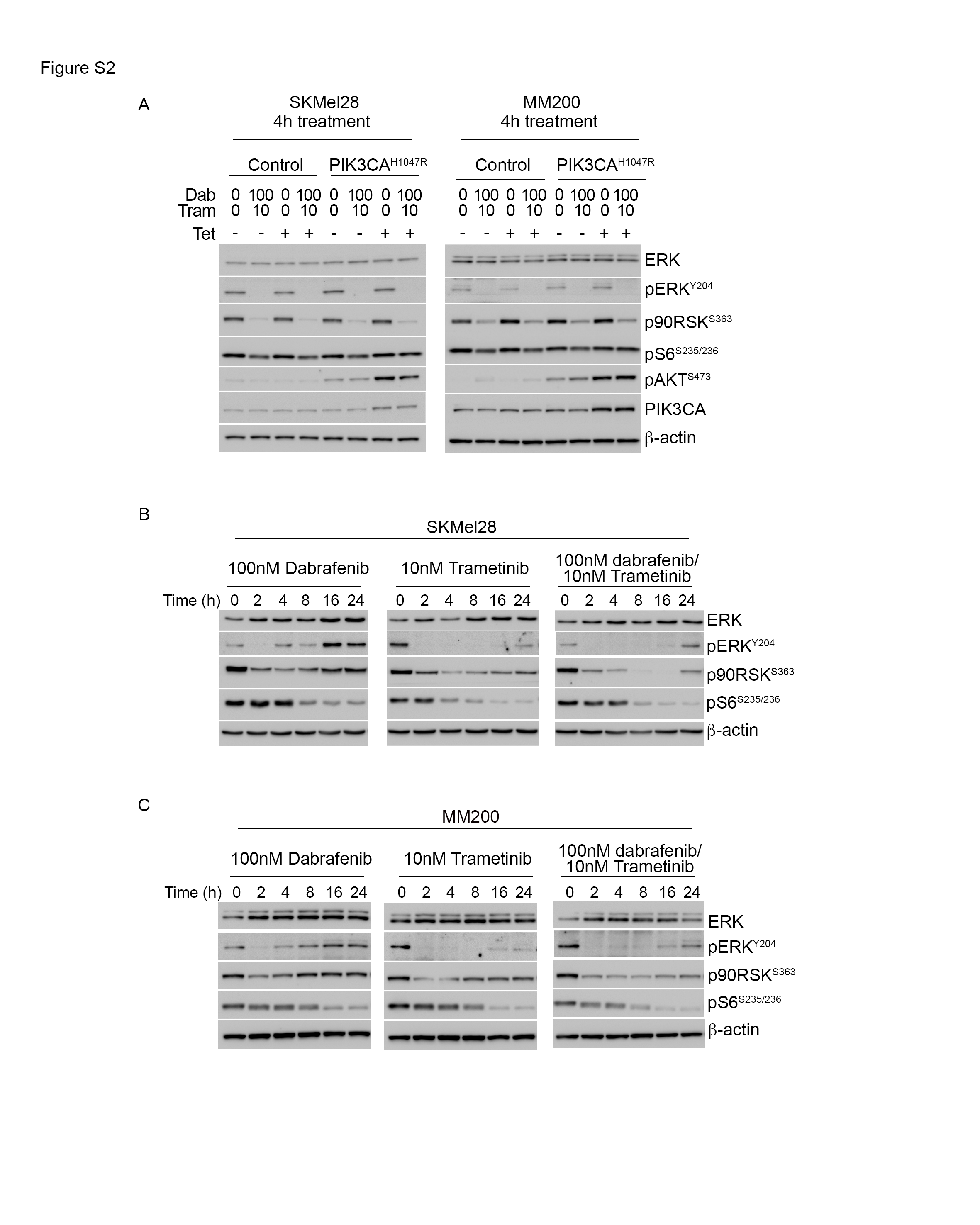

Supplement: Supplementary file 3 — Figure S2 [file 41389_2018_81_MOESM3_ESM.tif]

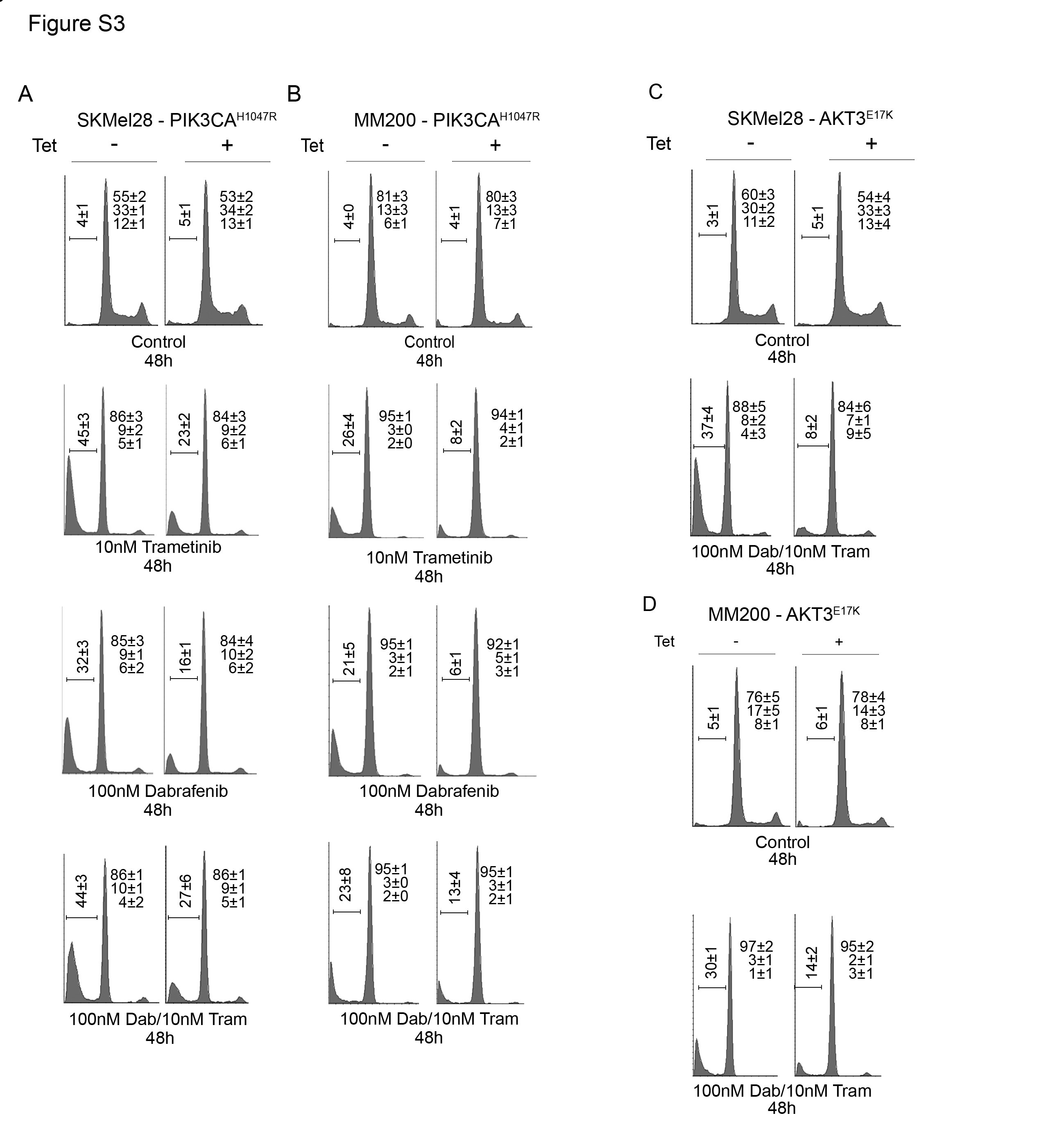

Supplement: Supplementary file 4 — Figure S3 [file 41389_2018_81_MOESM4_ESM.tif]

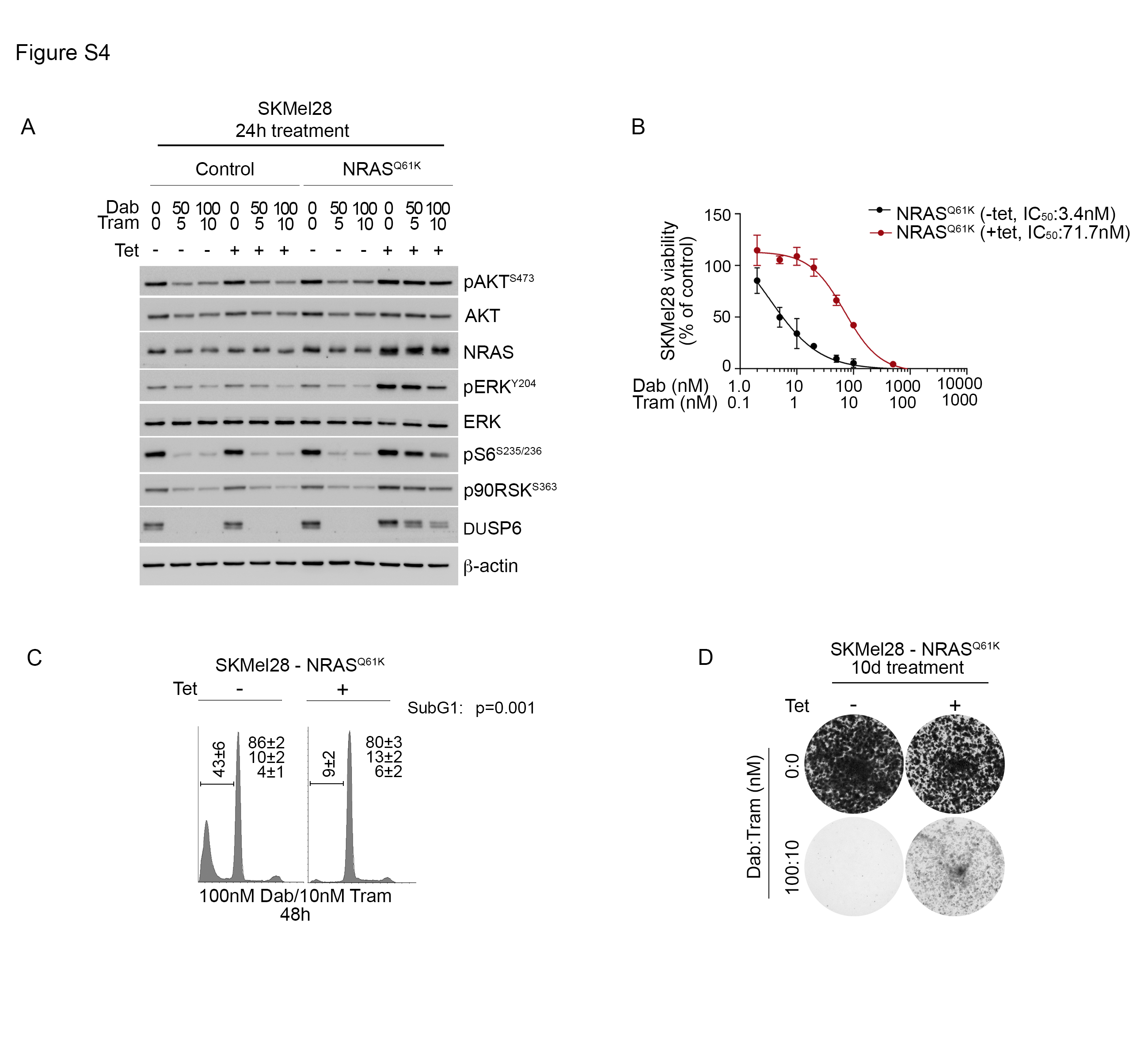

Supplement: Supplementary file 5 — Figure S4 [file 41389_2018_81_MOESM5_ESM.tif]

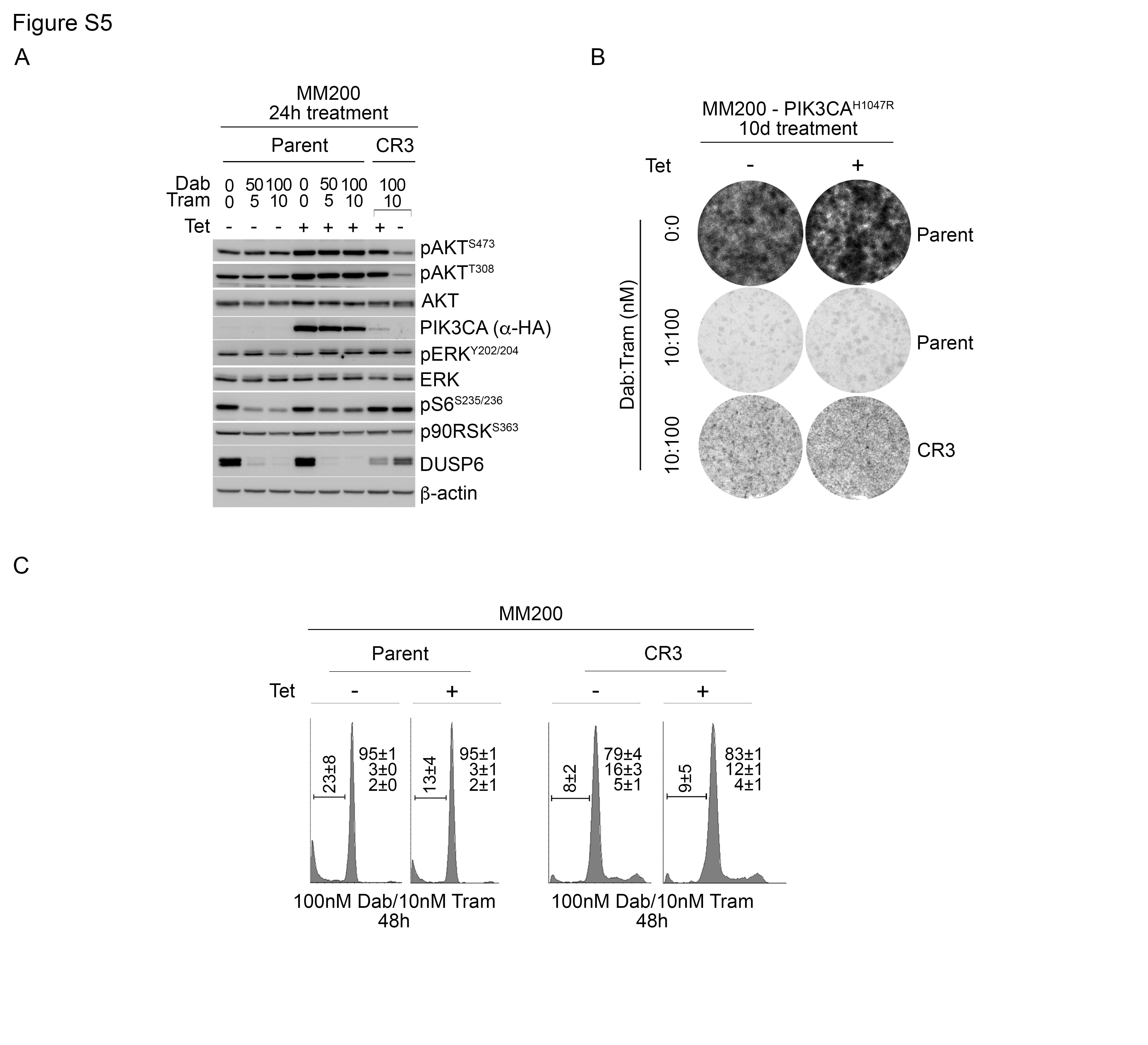

Supplement: Supplementary file 6 — Figure S5 [file 41389_2018_81_MOESM6_ESM.tif]

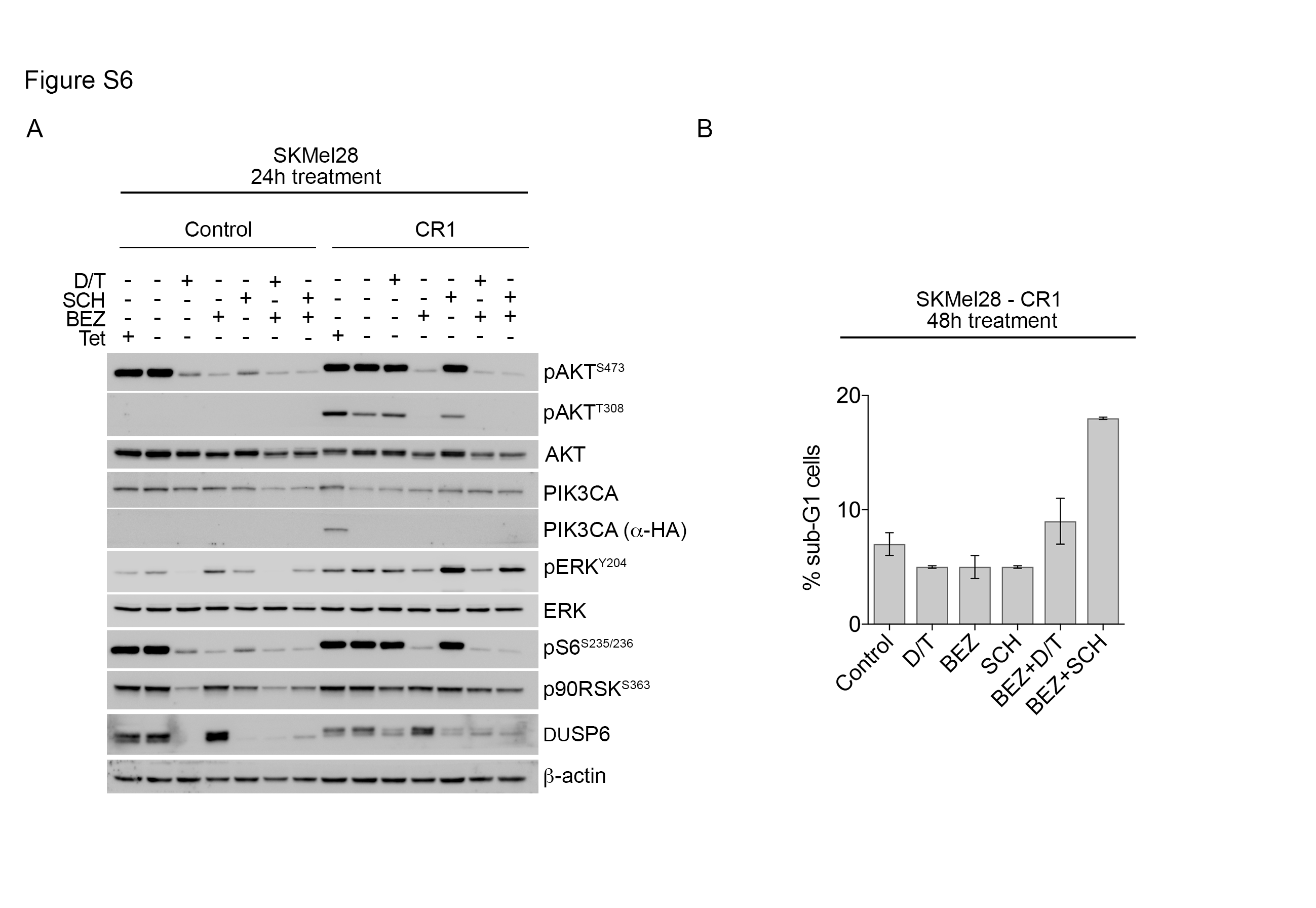

Supplement: Supplementary file 7 — Figure S6 [file 41389_2018_81_MOESM7_ESM.tif]
